# Posttraumatic stress disorder under ongoing threat: a review of neurobiological and neuroendocrine findings

**DOI:** 10.3402/ejpt.v7.30915

**Published:** 2016-08-09

**Authors:** Iro Fragkaki, Kathleen Thomaes, Marit Sijbrandij

**Affiliations:** 1 Behavioural Science Institute, Radboud University Nijmegen, Nijmegen, The Netherlands; 2 VU University Medical Center, VU University Amsterdam, Amsterdam, The Netherlands; 3 Department of Clinical Psychology, EMGO Institute for Health and Care Research, VU University Amsterdam, Amsterdam, The Netherlands

**Keywords:** PTSD, ongoing threat, neurobiology, neuroendocrinology, cortisol, hippocampus, amygdala

## Abstract

**Background:**

Although numerous studies have investigated the neurobiology and neuroendocrinology of posttraumatic stress disorder (PTSD) after single finished trauma, studies on PTSD under ongoing threat are scarce and it is still unclear whether these individuals present similar abnormalities.

**Objective:**

The purpose of this review is to present the neurobiological and neuroendocrine findings on PTSD under ongoing threat. Ongoing threat considerably affects PTSD severity and treatment response and thus disentangling its neurobiological and neuroendocrine differences from PTSD after finished trauma could provide useful information for treatment.

**Method:**

Eighteen studies that examined brain functioning and cortisol levels in relation to PTSD in individuals exposed to intimate partner violence, police officers, and fire fighters were included.

**Results:**

Hippocampal volume was decreased in PTSD under ongoing threat, although not consistently associated with symptom severity. The neuroimaging studies revealed that PTSD under ongoing threat was not characterized by reduced volume of amygdala or parahippocampal gyrus. The neurocircuitry model of PTSD after finished trauma with hyperactivation of amygdala and hypoactivation of prefrontal cortex and hippocampus was also confirmed in PTSD under ongoing threat. The neuroendocrine findings were inconsistent, revealing increased, decreased, or no association between cortisol levels and PTSD under ongoing threat.

**Conclusions:**

Although PTSD under ongoing threat is characterized by abnormal neurocircuitry patterns similar to those previously found in PTSD after finished trauma, this is less so for other neurobiological and in particular neuroendocrine findings. Direct comparisons between samples with ongoing versus finished trauma are needed in future research to draw more solid conclusions before administering cortisol to patients with PTSD under ongoing threat who may already exhibit increased endogenous cortisol levels.

**Highlights of the article:**

- We reviewed the neurobiological and neuroendocrine findings in PTSD under ongoing threat.
- PTSD under ongoing threat demonstrated similar brain abnormalities as PTSD after finished trauma.
- Several studies found increased cortisol levels in PTSD under ongoing threat.
- Hydrocortisone administration might not be beneficial for individuals with PTSD under ongoing threat with elevated endogenous cortisol levels.
- Direct comparisons between ongoing versus finished trauma are needed to provide robust conclusions and clinical recommendations.

Traumatic experiences refer to direct or indirect exposure to actual or threatened death, serious injury, or sexual violation (American Psychiatric Association [APA], 2013). Scientific evidence suggests that trauma exposure is related to the development of several psychiatric disorders, most notably posttraumatic stress disorder (PTSD), which is characterized by symptoms of re-experiencing, avoidance, negative cognitions/mood, and arousal (APA, 2013).

After trauma, PTSD develops in approximately 9% of individuals (Breslau et al., 1998) but rates vary significantly and are usually higher during ongoing trauma (e.g., 14–92% in intimate partner violence [IPV]) (Atwoli, Stein, Koenen, & McLaughlin, 2015; Dillon, Hussain, Loxton, & Rahman, 2013; Lagdon, Armour, & Stringer, 2014) than after single trauma. Despite these high prevalence rates in populations affected by ongoing threat, studies examining PTSD under ongoing threat are relatively scarce. This may be explained by the fact that studies on ongoing trauma have to be carried out under challenging circumstances (e.g., war areas) or individuals experiencing ongoing domestic violence may be reluctant to participate in research.

One reason why it is important to study PTSD under ongoing threat is that it is yet unclear to what extent the clinical manifestation of PTSD and associated responses in ongoing trauma differ from that of PTSD after finished trauma. Therefore, there is no consensus on how PTSD symptoms during ongoing trauma may be addressed. Many experts assume that for successful PTSD treatment, patients need to be in a safe and stable situation (Warshaw, Sullivan, & Rivera, 2013), which may lead to withholding or postponing evidence-based PTSD treatments, such as trauma-focused cognitive behavioral therapy (CBT) (National Collaborating Centre for Mental Health UK, 2005). Other experts, however, have started treatment even under challenging and threatening circumstances, for instance in a refugee camp (Acarturk et al., 2015, 2016). These randomized controlled trials found beneficial effects of eye movement desensitization and reprocessing (EMDR) in reducing PTSD symptoms and depression in adult Syrian refugees with PTSD located in a Turkish refugee camp on the Syrian border. Furthermore, recent efforts have been carried out to augment the effect of exposure-based treatment for PTSD with pharmacological agents such as hydrocortisone, D-cycloserine, MDMA, and propranolol, which might enhance extinction learning and reconsolidation (for a review see De Kleine, Rothbaum, & Van Minnen, 2013). A recent randomized controlled trial (RCT) examined the effect of hydrocortisone in veterans with PTSD and found that hydrocortisone augmentation enhanced the effectiveness of prolonged exposure, possibly through the effect of hydrocortisone on extinction memory, the increase of treatment retention, and the “normalization” of glucocorticoid sensitivity (Yehuda et al., 2015). However, it is not clear how these pharmacological augmentation strategies would benefit PTSD patients under ongoing threat because they are likely to show differential neuroendocrine responses than individuals with finished trauma. As such, there is no empirical support or neurobiological evidence to underpin current treatment recommendations for PTSD under ongoing threat. Additionally, more knowledge about the neurobiology of PTSD under ongoing threat may be relevant to the development of new psychological or pharmacological treatment strategies specifically tailored to individuals with ongoing trauma.

Extensive research has been conducted on structural (see meta-analyses of Karl et al., 2006; Kitayama, Vaccarino, Kutner, Weiss, & Bremner, 2005; Meng et al., 2014; Li et al., 2014; O'Doherty, Chitty, Saddiqui, Bennett, & Lagopoulos, 2015) and functional neurobiological correlates of PTSD (see meta-analyses of Etkin & Wager, 2007; Hayes, Hayes, & Mikedis, 2012; Patel, Spreng, Shin, & Girard, 2012; Ramage et al., 2013; Sartory et al., 2013; Stark et al., 2015), but it has primarily focused on PTSD after traumatic events that are finished. Structural and functional abnormalities have been found in prefrontal and limbic areas, which are involved in cognitive and emotional processing.

Specifically, the amygdala is involved in emotional processing, acquisition, expression, and regulation of fear and traumatic memories, as well as fear conditioning and generalization (Duvarci & Pare, 2014; Kim et al., 2011; Marek, Strobel, Bredy, & Sah, 2013). Although previous studies found lower amygdala volume in PTSD patients (Karl et al., 2006), a recent meta-analysis found decreased amygdala volume in PTSD patients compared to non-exposed healthy controls but not compared to trauma-exposed individuals without PTSD, suggesting that decreased amygdala volume is related to trauma exposure and not PTSD (O'Doherty et al., 2015). PTSD patients exhibit heightened fear response and hypervigilance to environmental stimuli (APA, 2013; Ehlers & Clark, 2000), which is associated with increased amygdala activity. PTSD patients exhibit hyperactivation of amygdala in response to trauma-related and general affective stimuli that reinforces the vivid nature of intrusions and hyperarousal (Hayes et al., 2012; Sartory et al., 2013; Stark et al., 2015). However, another meta-analysis revealed that the hyperactivation of amygdala was observed only in comparison to non-exposed healthy controls and not to trauma-exposed controls without PTSD, indicating that this association might be related to trauma exposure and not PTSD (Patel et al., 2012). A comparison of studies on PTSD, social anxiety disorder, and specific phobia demonstrated that the hyperactivation of amygdala and insula were more salient in social anxiety disorder and specific phobia than PTSD (Etkin & Wager, 2007).

PTSD patients also present memory impairments and hippocampal dysfunction, which is associated with abnormal sensation-based and contextual representations of a traumatic event (Acheson, Gresack, & Risbrough, 2012; Brewin, Gregory, Lipton, & Burgess, 2010). The hippocampus is involved in the formation of long-term, declarative memory and memory processing (Battaglia, Benchenane, Sirota, Pennartz, & Wiener, 2011), and it is significantly smaller in PTSD patients compared to non-exposed and trauma-exposed healthy individuals (Karl et al., 2006; Kitayama, et al., 2005; O'Doherty et al., 2015). In addition, PTSD patients exhibit abnormal brain activity in hippocampal and parahippocampal regions, which might affect the formation of traumatic memories (Patel et al., 2012; Stark et al., 2015).

Furthermore, structural and functional brain abnormalities in prefrontal regions have been implicated in PTSD. The anterior cingulate cortex (ACC) is involved in the regulation of negative feedback of the hypothalamic–pituitary–adrenal (HPA) axis, regulation of emotions, and behavioral inhibition; the prefrontal cortex (PFC) is associated with complex cognitive processes (executive function, working memory, decision making), from which the medial PFC (mPFC) is involved in extinction of conditioned fear and emotion regulation (Etkin & Wager, 2007; Giustino & Maren, 2015; Zoladz & Diamond, 2013). Meta-analyses of structural studies yielded reduced volume of the ACC in PTSD patients compared to non-exposed and trauma-exposed controls (Karl et al., 2006; Meng et al., 2014; O'Doherty et al., 2015). Evidence from functional studies showed decreased activity of ACC, mPFC, and left inferior frontal cortex in PTSD patients (Hayes et al., 2012; Patel et al., 2012; Stark et al., 2015). It is proposed that the ACC and PFC are involved in the pathophysiology of PTSD via a failure to inhibit amygdala hyperactivation in response to environmental stimuli that are perceived as threatening. Overall, these brain areas play a role in the pathophysiology of PTSD because of their involvement in the regulation of emotions, fear responses, and memory formation that are impaired in individuals with PTSD.

These brain structures do not function independently, but take part in large-scale brain networks: the default mode network (DMN), the central executive network (CEN), and the salience network (SN) (Hayes et al., 2012; Patel et al., 2012; Ramage et al., 2013; Sartory et al., 2013). The DMN includes the mPFC, posterior cingulate cortex, lateral and medial temporal lobes, and posterior inferior parietal lobule and is mainly involved in self-referential thinking (Andrews-Hanna, 2012; Spreng, Mar, Kim., 2009). The CEN includes the dorsolateral PFC and lateral parietal cortex and it is related to attentional control and working memory (Menon, 2011). The SN is anchored in the dorsal ACC and fronto-insular cortex and closely connected to the amygdala, thalamus, ventral basal ganglia and is involved in autonomic and emotion regulation and reward processing (Patel et al., 2012). PTSD has been associated with hypoactivation of the DMN and CEN, and hyperactivation of the SN (Aupperle et al., 2016; Hayes et al., 2012; Lanius, Frewen, Tursich, Jetly, & McKinnon, 2015; Patel et al., 2012; Ramage et al., 2013; Sartory et al., 2013). These findings confirmed the abnormalities in amygdala, hippocampus, PFC, and ACC in PTSD, but they also revealed abnormalities in other brain areas incorporating all of them in larger brain networks. A recent review proposed specific associations between the abnormalities in these brain networks and the clinical symptoms of PTSD (Lanius et al., 2015). The authors suggested that cognitive dysfunction is related to CEN, hypo- and hyperarousal/interoception is related to SN, and identity disturbances, such as depersonalization, are related to DMN (Lanius et al., 2015). Overall, the neurocircuitry model of PTSD suggests hyperresponsivity of amygdala, hyporesponsivity of mPFC, and an inability of mPFC and hippocampus to inhibit the amygdala, as well as hypoactivation of the DMN and CEN, and hyperactivation of the SN. However, it is still unknown whether PTSD under ongoing threat presents the same abnormal patterns.

Regarding the neuroendocrine model of PTSD, trauma exposure is associated with dysregulated HPA activity and abnormal cortisol patterns (Daskalakis, Lehrner, & Yehuda, 2013; Klaassens et al., 2012; Meewisse, Reitsma, De Vries, Gersons, & Olff, 2007; Miller, Chen, & Zhou, 2007; Morris, Compas, & Garber, 2012; Wingenfeld & Wolf, 2014; Zoladz & Diamond, 2013). Moreover, during chronic stress cortisol levels are elevated but with the passage of time HPA activity decreases and cortisol secretion goes below the normal levels. Importantly, with a stressor still present, cortisol levels are significantly higher across the day and the daily cortisol output is higher compared to controls (Miller et al., 2007).

Studies showed mixed results reporting increased (Miller et al., 2007), decreased (Morris et al., 2012) or no difference (Klaassens et al., 2012; Meewisse et al., 2007) in cortisol levels for PTSD patients compared to trauma-exposed and non-trauma-exposed controls. This inconsistency may be due to methodological differences, such as different time points of cortisol measurements (Klaassens et al., 2012; Morris et al., 2012; Zoladz & Diamond, 2013). The meta-analysis by Miller et al. (2007) supported that PTSD patients exhibited higher cortisol levels in afternoon/evening, lower daily output of cortisol, and lower levels of post-dexamethasone cortisol than healthy trauma-exposed individuals. A more recent meta-analysis presented evidence for lower morning and afternoon cortisol levels as well as lower daily cortisol output in PTSD patients compared to healthy non-exposed controls (Morris et al., 2012). PTSD patients also exhibited enhanced cortisol suppression compared to healthy controls but not compared to trauma-exposed individuals without PTSD. Importantly, for PTSD, afternoon cortisol levels, daily output, and cortisol suppression were lower when more time had passed since the trauma (Morris et al., 2012).

In contrast, other meta-analyses did not find systematic differences in cortisol levels between PTSD patients and trauma-exposed or non-exposed healthy controls (Klaassens et al., 2012; Meewisse et al., 2007). The meta-analysis by Klaassens et al. (2012) showed that individuals exposed to adulthood trauma with or without PTSD did not exhibit abnormal cortisol levels compared to healthy controls, but trauma exposure led to increased cortisol suppression after dexamethasone administration. However, this meta-analysis did not examine potential differences between time points of cortisol measurements. The meta-analysis by Meewisse et al. (2007) also yielded no differences in cortisol between PTSD patients and controls, but subgroup analyses revealed that lower cortisol levels were associated with female gender, history of physical or sexual abuse, afternoon measurements, and trauma exposure (Meewisse et al., 2007).

Recent studies have examined hair cortisol as an indicator of chronic cortisol concentration but the findings in PTSD samples are scarce, inconsistent, and methodologically diverse (Vives et al., 2015). It is interesting to note though that hair cortisol was higher in PTSD patients who were living in a civil war area in Uganda (Steudte et al., 2011), but lower in PTSD patients who experienced an earthquake (Luo et al., 2012) or other various traumatic events (e.g., accidents, sexual assaults, death of a loved one) (Steudte et al., 2013). Taken together, we hypothesized that individuals with PTSD under ongoing threat would exhibit higher cortisol levels in afternoon/evening and higher daily output of cortisol based on the finding that in the presence of ongoing stressors cortisol levels tend to be higher and that the more time passed since the trauma, the lower the cortisol levels (Miller et al., 2007; Morris et al., 2012).

Inconsistencies in the abovementioned neurobiological and neuroendocrine findings might be explained by other factors. First, type and time onset of trauma exposure might influence the neurobiological and neuroendocrine alterations (Karl et al., 2006; Miller et al., 2007; Ozer, Best, Lipsey, & Weiss, 2003; Zoladz & Diamond, 2013). Second, PTSD is often manifested with comorbid disorders, such as depression and substance abuse disorders (Caramanica, Brackbill, Liao, & Stellman, 2014; Debell et al., 2014; Spinhoven, Penninx, Van Hemert, De Rooij, & Elzinga, 2014), but most of the studies did not control for comorbidity. Third, PTSD patients with dissociative symptoms present substantial differences than patients without dissociative symptoms, which led to the addition of a dissociative subtype of PTSD in DSM-5 (APA, 2013; Stein et al., 2013; Wolf, 2013). Although the most frequently present “intrusive” PTSD is characterized by emotional undermodulation exhibiting low PFC activity and increased amygdala activation as described above, the dissociative subtype of PTSD is characterized by emotional overmodulation and exhibits increased PFC and decreased limbic activity (Lanius et al., 2010). Nonetheless, the majority of previous studies did not account for that factor and it is possible that some of the observed inconsistencies are because of these different brain patterns. Fourth, there is some evidence that additional problems in emotional regulation, negative self-concept, and interpersonal relationships give a slightly different neurobiological pattern than the dissociative subtype of PTSD (Cloitre, Garvert, Weiss, Carlson, & Bryant, 2015; Lanius, Bluhm, & Frewen, 2011; Marinova & Maercker, 2015). Whereas the dissociative subtype shows the reversed pattern of “intrusive” PTSD with increased PFC and low limbic activity, in complex PTSD increased PFC activity seems not to lead to overmodulation, because limbic activity stays increased too (Marinova & Maercker, 2015; Thomaes et al., 2009, 2010, 2013).

The aim of this review is to present an overview of studies that investigated the neurobiology and neuroendocrinology of PTSD in adults under ongoing threat, including victims of IPV, police officers, and fire fighters whose everyday environment is replete with potentially traumatic and life-threatening situations. We propose that because of the frequent and ongoing experience of potentially traumatic events, these individuals might present different neurobiological and neuroendocrine patterns than the patterns describes in recent meta-analyses on individuals with PTSD after finished trauma.

## Method

**Fig. 1:**
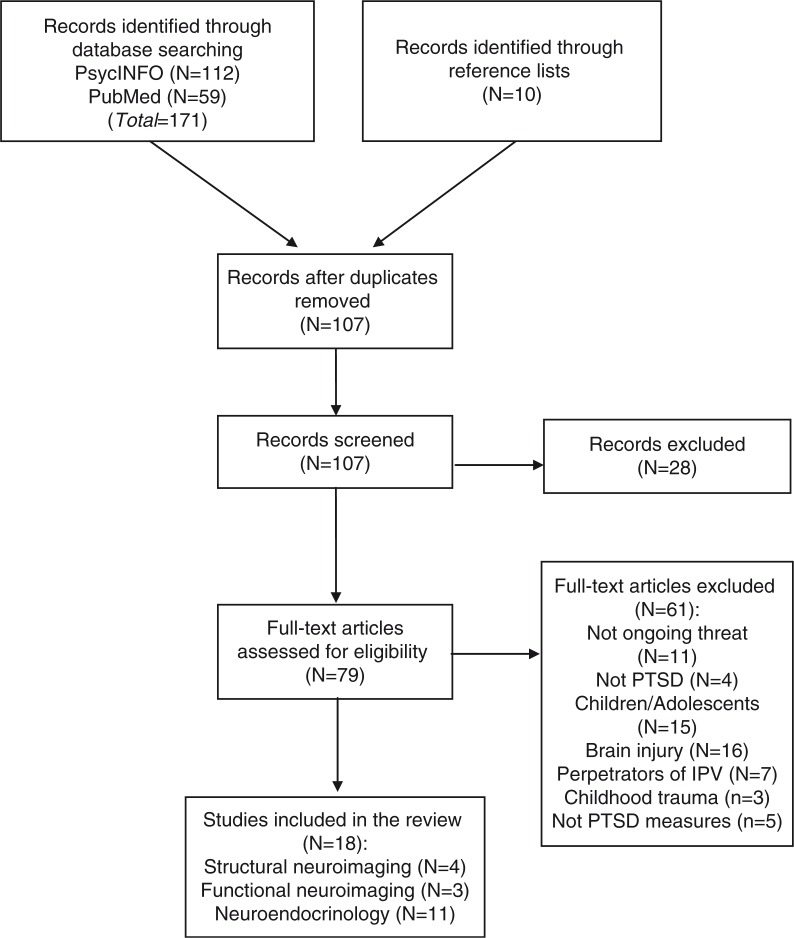
Flow diagram of studies on PTSD under ongoing threat.

We performed an electronic search in the databases PsycINFO and PubMed using the key terms IPV, domestic violence, police officers, battered women, fire fighters, ongoing threat, ongoing trauma, continuous trauma, continuous threat, terrorism, war zones, child soldiers in combination with brain, neurobiology, amygdala, hippocampus, hippocampal, cortex, functional magnetic resonance imaging (fMRI), PET, neuroimaging, cortisol, and HPA-axis. We combined all of the following search terms: posttraumatic stress disorder and population (intimate partner violence, police officers, battered women, fire fighters, etc.) with: neurobiology (brain, neurobiology, amygdala, hippocampus, hippocampal, etc.) or neuroendocrinology (cortisol, HPA-axis). Examples of search streams are (1) “posttraumatic stress disorder” AND “police officers” AND “amygdala,” (2) “posttraumatic stress disorder” AND “intimate partner violence” AND “cortisol.” The search was conducted for articles from 1990 to 2016 published in English. The inclusion criteria were the following: (1) adult populations of victims of IPV, war trauma, police officers, and fire fighters, (2) ongoing character of trauma: IPV victims with recent incidents, in an abusive relationship at the time of the study or living in a shelter, and police officers and fire fighters in active duty, (3) outcome measure of PTSD symptoms, and (4) a comparison group of healthy controls or trauma-exposed individuals with or without PTSD. Studies with victims of IPV who were living in shelters were included, because these women were still living in fear of their abuser and were afraid to live on their own. Figure 1 demonstrates the flow diagram of the search starting with 171 studies in total. After removing duplicates and excluding studies that did not meet the inclusion criteria, we included 18 studies. No studies on individuals living in conflict zones were found. These 18 studies comprised 2,989 participants (2,193 males, 796 females), 4 studies on structural neuroimaging, 3 on functional neuroimaging, and 11 on neuroendocrinology.

## Results

We retrieved four structural studies on brain volume of hippocampus and amygdala, and three functional studies on brain activity of PFC, amygdala, and insula (Tables 1 and 2).

**Table 1:** Structural neuroimaging studies on PTSD under ongoing threat

Study	Participants	Gender	Type of study	Findings
Fennema-Notestine et al. (2002)	*N*=39 11 IPV with PTSD 11 IPV without PTSD 17 control persons	All females	MRI (whole brain image series)	No differences in amygdala, hippocampal and parahippocampal gyrus volume
Lindauer, Vlieger, et al. (2004); Lindauer et al. (2006)	*N*=28 police officers 14 with PTSD 14 without PTSD	16 males 12 females	MRI (volumes of hippocampus, amygdala, parahippocampal gyrus)	Smaller total and left hippocampal volume in PTSD No differences in amygdala and parahippocampal gyrus volume
Shucard et al. (2012)	*N*=15 police officers 6 with PTSD symptoms 9 without PTSD	All males	MRI (subcortical segmentation)	Re-experiencing negatively associated with right amygdala, left globus pallidus, left thalamus volume

IPV: intimate partner violence; PTSD: posttraumatic stress disorder; MRI: magnetic resonance imaging.

**Table 2:** Functional neuroimaging studies on PTSD under ongoing threat

Study	Participants	Gender	Type of study	Findings PTSD vs. control group
Lindauer, Booij, et al. (2004)	*N*=30 police officers 15 with PTSD 15 without PTSD	18 males 12 females	SPECT with trauma versus neutral scripts	Decreased activation of left medial frontal gyrus and Broca's area No differences in amygdala activation
Simmons et al. (2008)	*N*=30 15 IPV–PTSD 15 non traumatized control persons	All females	fMRI with an anticipatory task of positive and negative stimuli	Higher activation of right anterior/middle insula Decreased connectivity of insula and amygdala
Peres et al. (2011)	*N*=36 police officers 24 with partial PTSD 12 without PTSD	All males	fMRI with an acoustic-cue paradigm with three pleasant, neutral, and traumatic memories	Higher left-amygdala activity Lower mPFC activity

IPV: intimate partner violence; PTSD: posttraumatic stress disorder; fMRI: functional magnetic resonance imaging; mPFC: medial prefrontal cortex; SPECT: single-photon emission computed tomography.

### Structural neuroimaging studies

#### Hippocampus

Four studies with three different samples (one in IPV victims, two in police officers) measured the hippocampal volume as presented in Table 1. The first MRI study showed no significant difference in the volume of the bilateral hippocampus or parahippocampal gyrus between the groups in 11 victims of IPV with PTSD, 11 victims of IPV without PTSD, and 17 non-victimized control persons (Fennema-Notestine, Stein, Kennedy, Archibalb, & Jernigan, 2002). An MRI study among 28 police officers (14 with PTSD, 14 without PTSD, excluding individuals with comorbid Major Depressive Disorder [MDD] or substance-related disorders) found a smaller total and left hippocampal volume in the PTSD group compared to controls, but no differences in the volume of parahippocampal gyrus volume (Lindauer, Olff, Van Meijel, Carlier, & Gersons 2006; Lindauer, Vlieger, et al., 2004). It was also found that re-experiencing symptoms in PTSD patients were associated with reduced left hippocampal volume (Lindauer et al., 2006; Lindauer, Vlieger, et al., 2004). A similar trend for a relationship between elevated re-experiencing symptoms and reduced hippocampal volume was found in another MRI study in 15 police officers (a small sample with only 6 PTSD patients and 9 persons without PTSD or subsyndromal PTSD symptoms) (Shucard et al., 2012).

#### Amygdala

The above-mentioned studies indicated that amygdala volume was not significantly smaller in victims of IPV or police offenders with PTSD compared to controls (Fennema-Notestine et al., 2002; Lindauer, Vlieger, et al., 2004). However, a trend for an association between re-experiencing symptoms and smaller amygdala volume was found in police officers with PTSD in the study by Shucard et al. (2012).

### Functional neuroimaging studies

Three fMRI studies investigated brain activity in PTSD under ongoing threat focusing on amygdala, insula, hippocampus, and PFC (Table 2). An fMRI study included 15 IPV victims with PTSD and 25 non-traumatized controls and investigated insula activation and functional connectivity in response to an anticipatory task of positive and negative stimuli (Simmons et al., 2008). The IPV–PTSD group demonstrated significantly higher activation of right anterior/middle insula compared to controls in anticipation of negative stimuli. Furthermore, the functional connectivity of bilateral anterior/right, anterior/middle insula and bilateral amygdala were significantly weaker in IPV–PTSD group compared to controls. Another study investigated the neuronal circuitry in response to neutral and trauma-based script-driven imagery in 30 police officers, 15 with PTSD and 15 without PTSD with SPECT Imaging (Lindauer, Booij, et al., 2004). The results revealed decreased activation of medial frontal gyrus, left medial frontal gyrus, and Broca's area and increased activation of the right cuneus in response to trauma versus neutral scripts in PTSD compared to controls, but no differences in amygdala activation.

Finally, there is evidence of specific associations between PTSD symptoms or PTSD severity and brain activity. An fMRI study on police officers (24 with PTSD symptoms of re-experiencing and hyperarousal, 12 without PTSD) examined brain activity of amygdala and PFC during an acoustic-cue paradigm with pleasant, neutral, and traumatic memories (Peres et al., 2011). The results indicated that the PTSD group demonstrated increased activation of left amygdala and decreased activation of mPFC compared to controls during retrieval of traumatic memories. However, this study included only individuals with re-experiencing and hyperarousal symptoms who did not meet all the DSM-IV criteria for PTSD and thus these findings refer to these specific PTSD symptoms. In line with this association, the study by Simmons et al. (2008) also found a positive association between hyperarousal symptoms and activity in left anterior insula.

### Neuroendocrine studies

We extracted 11 studies that investigated cortisol levels in relation to PTSD under ongoing threat. There was a great variability in timing, type, and number of cortisol measurements among the studies (Table 3).

**Table 3:** Neuroendocrine studies on PTSD under ongoing threat

Study	Sample	Gender	Time of measurement	Findings
Pico-Alfonso et al. (2004)	*N*=162 30 IPV–PTSD 86 IPV without PTSD 46 control persons	All females	2 samples for 4 consecutive days: 8 am and 8 pm	No association between cortisol levels and PTSD IPV was associated with increased evening cortisol levels
Griffin et al. (2005)	*N*=64 15 IPV–PTSD 27 IPV–PTSD+MDD 8 IPV without diagnosis 14 non-traumatized control persons	All females	2 samples: day 1, day 2 Dexamethasone (0.5 mg) administration	PTSD and PTSD+MDD had lower baseline cortisol levels compared to control persons PTSD showed greater cortisol suppression after dexamethasone compared to PTSD+MDD and control persons
Neylan et al. (2005)	*N*=30 police officers 5 with PTSD 25 without PTSD	24 males 6 females	4 samples: 1, 30, 45, 60 min after awakening Dexamethasone (0.5 mg) administration	PTSD severity was related to lower levels of baseline cortisol No association between PTSD and post-dexamethasone cortisol
Inslicht et al. (2006)	*N*=49 29 IPV–PTSD 20 IPV without PTSD	All females	4 samples; 1, 4, 9, 11 hours after awakening	Higher cortisol levels across the day in IPV–PTSD
Lindauer et al. (2006)	*N*=24 police officers 12 with PTSD 12 without PTSD	14 males 10 females	3 samples: early morning, 4 pm, bedtime	Higher morning cortisol levels in PTSD
Johnson et al. (2008)	*N*=52 32 IPV–PTSD 20 IPV without PTSD	All females	4 samples: upon awakening, 30, 45, 60 min after	PTSD group had higher cortisol levels More chronic abuse was related to lower waking cortisol level
Witteveen et al. (2010)	*N*=1,880 police officers and fire fighters 98 with PTSD 1,782 without PTSD	1,703 males 177 females	1 saliva sample at morning or noon or afternoon	No association between cortisol levels and PTSD More negative life events were associated with lower cortisol levels
Austin-Ketch et al. (2011)	*N*=100 police officers 35 with PTSD symptoms 65 without PTSD symptoms	58 males 42 females	13 samples: upon awakening, 15, 30, 45, 60 min after, prior to lunch/dinner, bedtime	Trend for higher cortisol levels in PTSD
Inslicht et al. (2011)	*N*=296 police officers 9 with partial or full PTSD 287 without PTSD	254 males 42 females	2 samples (baseline, 12, 24, 36 months): upon awakening, 30 min after	Waking cortisol was not associated with PTSD symptoms
Pineles et al. (2013)	*N*=60 police officers and fire fighters	55 males 5 females	Immediately after waking cortisol	No association between PTSD and cortisol
Pinna et al. (2014)	*N*=104 68 IPV–PTSD (43 +MDD) 36 IPV without PTSD	All females	4 samples: upon awakening, 30, 45, 60 min after	Higher waking cortisol in PTSD+MDD compared to controls PTSD only was not related to waking cortisol

IPV: intimate partner violence; PTSD: posttraumatic stress disorder; MDD: major depressive disorder.

Two studies compared daily cortisol output in PTSD patients to that in controls. A study on 29 women with IPV–PTSD and 20 with IPV without PTSD found an association between PTSD and higher average daily cortisol output (Inslicht et al., 2006). The results remained significant even after controlling for age, depression, latency of abuse, and PTSD severity underscoring the independent effect of PTSD on cortisol levels. Another study recruited 100 police officers, 35 with PTSD symptoms and 65 without PTSD symptoms, and measured cortisol levels across the day for 3 consecutive days (Austin-Ketch et al., 2011). The results were mixed presenting differences between cortisol levels and PTSD severity. Specifically, mild PTSD symptoms were significantly associated with higher diurnal cortisol output compared to subclinical PTSD symptoms, but the differences between subclinical and high PTSD symptoms were not significant.

The morning, afternoon, and evening cortisol levels were investigated in three studies. The study by Lindauer et al. (2006) mentioned above also investigated the relation between morning, afternoon, and evening cortisol levels and PTSD in 12 police officers with PTSD and 12 police officers without PTSD. They found that individuals with PTSD had higher early morning cortisol levels than trauma-exposed controls. Morning and evening cortisol levels were also examined for four consecutive days in 162 participants, 30 IPV–PTSD with comorbid depression, 86 IPV without PTSD, and 46 non-traumatized controls (Pico-Alfonso, Garcia-Linares, Celda-Navarro, Herbert, & Martinez, 2004). There was no association between PTSD and cortisol levels, but IPV was related to elevated evening cortisol levels (Pico-Alfonso et al., 2004). It is noteworthy that the high levels of comorbid depression in the sample did not allow for a reliable independent measurement of the effect of PTSD on cortisol. Furthermore, a large study with 1,880 police officers and fire fighters investigated morning and afternoon cortisol levels (Witteveen et al., 2010). The sample included professionals who had experienced a specific disaster in 1992, professionals who had experienced the disaster and were still in active duty, and professionals who were not involved in the disaster. The results showed no significant association between PTSD and cortisol levels, although not all the participants were in active duty at the time of the study (Witteveen et al., 2010). However, a negative correlation between cortisol and negative life events was revealed, suggesting that trauma exposure and not PTSD is related to lower cortisol levels.

In addition, the cortisol awakening response (CAR) was the focus of four studies. Johnson, Delahanty, and Pinna (2008) examined CAR in 32 women with IPV–PTSD and 20 women with IPV without PTSD. PTSD severity was related to an increased CAR, whereas chronic abuse was related to lower CAR. Moreover, the results yielded a flattened waking cortisol curve in women with IPV without PTSD, a pattern associated with chronic stress. Another recent study measured CAR in 104 participants, 68 women with IPV–PTSD (43 of them with comorbid MDD, 12 with MDD only, and 24 with IPV without PTSD or MDD) (Pinna, Johnson, & Delahanty, 2014). Participants with PTSD exhibited an increased CAR compared to those without PTSD. However, when accounting for comorbidity, the PTSD-only group did not differ significantly from the other groups on waking cortisol, whereas the MDD-only group had significantly increased CAR compared to controls. The authors concluded that comorbid MDD is responsible for the increased CAR that is usually reported in PTSD. Similarly, another study examined CAR in 37 police officers and fire fighters and yielded no significant association between PTSD symptoms and waking cortisol levels (Pineles et al., 2013). CAR was also not associated with PTSD in 296 police officers during academy training and after 12, 24, and 36 months (Inslicht et al., 2011). However, it is noteworthy that in this study only nine participants had partial or full PTSD after 36 months and, consequently, there was not enough power to detect significant changes.

Finally, two studies administered dexamethasone to examine cortisol suppression. The first one recruited 64 women, 15 with IPV–PTSD, 27 with IPV–PTSD +MDD, 8 with IPV without PTSD or MDD, and 14 controls (Griffin, Resick, & Yehuda, 2005). The results revealed that PTSD-only and PTSD with MDD groups exhibited lower baseline cortisol levels compared to the control groups and the PTSD-only group had significantly lower cortisol levels at day 2 (post-dexamethasone) compared to control groups. The second study examined pre- and post-dexamethasone cortisol in 30 police officers, 5 with PTSD and 25 without PTSD in relation to CAR (Neylan et al., 2005). They found that subjects with PTSD had significantly lower CAR pre-dexamethasone compared to controls, but there were no significant association between PTSD and post-dexamethasone cortisol levels.

## Discussion

The aim of this review was to present the neurobiological and neuroendocrine findings on PTSD under ongoing threat and examine whether PTSD under ongoing threat is characterized by abnormal patterns similar to those previously found in PTSD after finished trauma. We found no significant differences in brain volumes of amygdala and parahippocampal gyrus between PTSD under ongoing threat groups and trauma-exposed or healthy controls (Fennema-Notestine et al., 2002; Lindauer et al., 2006; Lindauer, Vlieger, et al., 2004), but a negative relation between re-experiencing symptoms and the volume of right amygdala was found in police officers with PTSD compared to police officers without PTSD (Shucard et al., 2012). The findings on the association between hippocampal volume and PTSD were conflicting: one study yielded no differences in hippocampal volume between PTSD and trauma-exposed or healthy controls (Fennema-Notestine et al., 2002), whereas two other found that police officers with PTSD had smaller hippocampal volume compared to those without PTSD (Lindauer et al., 2006; Lindauer, Vlieger, et al., 2004; Shucard et al., 2012).

Previous studies examining PTSD after finished trauma have supported a relationship between PTSD and smaller volumes of left amygdala, hippocampus, and ACC, although these results were not consistent across studies (Karl et al., 2006; Kitayama et al., 2005; Meng et al., 2014; O'Doherty et al., 2015). There is cumulative evidence that the association between PTSD and reduced amygdala volume is present only in comparison to healthy controls and not to trauma-exposed individuals, supporting that decreased amygdala volume is associated with trauma exposure and not PTSD (O'Doherty et al., 2015). The absence of an association between PTSD under ongoing threat and reduced amygdala is in line with these findings. However, the limited number of the studies examining brain structural abnormalities in individuals with PTSD under ongoing threat does not provide us with sufficient evidence to draw solid conclusions.

The reviewed fMRI studies on PTSD under ongoing threat revealed hyperactivation of amygdala and insula, hypoactivation of PFC, and decreased connectivity in response to negative stimuli (Lindauer, Booij, et al., 2004; Peres et al., 2011; Simmons et al., 2008). These findings are in concordance with the existing neurocircuitry model of PTSD that supports a pattern of hyperactivation of amygdala and hypoactivation of PFC and hippocampus (Hayes et al., 2012; Patel et al., 2012; Ramage et al., 2013; Sartory et al., 2013; Stark et al., 2015). However, hyperactivation of amygdala was observed in studies comparing PTSD patients to healthy controls (Simmons et al., 2008) and not when comparing PTSD patients to trauma-exposed police officers (Lindauer, Booij, et al., 2004). Therefore, hyperactivation of amygdala might be related to trauma exposure and not PTSD per se, which is in accordance with the findings in PTSD after single trauma (Etkin & Wager, 2007; Patel et al., 2012).

Concerning neuroendocrinology, the findings were highly inconsistent. Some studies revealed an association between PTSD and increased cortisol output, morning cortisol levels, and CAR compared to trauma-exposed controls (Austin-Ketch et al., 2011; Inslicht et al., 2006; Johnson et al., 2008; Lindauer et al., 2006), whereas others found no association (Inslicht et al., 2011; Pico-Alfonso et al., 2004; Pineles et al., 2013; Pinna et al., 2014; Witteveen et al., 2010). The two studies that examined dexamethasone administration suggested that PTSD was related to lower baseline cortisol levels, but only one of them found greater cortisol suppression after dexamethasone administration (Griffin et al., 2005; Neylan et al., 2005). It is noteworthy that four studies found an association between PTSD and *increased* cortisol levels, whereas two found a relation between PTSD and *decreased* baseline cortisol levels. Finally, three studies supported that lower cortisol response was associated with trauma exposure, but not with PTSD (Johnson et al., 2008; Pico-Alfonso et al., 2004; Witteveen et al., 2010). These findings are in agreement with the meta-analysis by Meewisse et al. (2007) that indicated an association between history of trauma and lower cortisol levels. Our hypothesis that PTSD under ongoing threat could be related to higher cortisol levels was supported by four studies, suggesting that the pattern of increased cortisol is more pronounced in PTSD than in healthy individuals under ongoing threat. However, the mixed results did not allow us to draw robust conclusions and further research is highly needed to disentangle this association.

### Limitations

The inconsistent findings may be explained by several factors that might have interfered and masked the association between neurobiological and neuroendocrine abnormalities and PTSD under ongoing trauma, such as history of childhood abuse, trauma exposure, comorbidity, presence of dissociative symptoms or complex PTSD, small sample sizes, methodological differences, and differences in number, type, and time of cortisol measurements. PTSD is associated with history of childhood abuse (Ozer et al., 2003) and high rates of comorbidity with depression and substance abuse disorders (Caramanica, Brackbill, Liao, & Stellman, 2014; Debell et al., 2014; Spinhoven, et al., 2014). It is therefore possible that the neurobiological and neuroendocrine abnormalities are related to these factors and not PTSD. Similarly, trauma exposure itself has been supported to be associated with neurobiological and neuroendocrine dysfunctions (Karl et al., 2006; Miller et al., 2007) and the dissociative subtype of PTSD has been found to present a distinctive pattern of brain abnormalities that should also be taken into account (Lanius et al., 2010). Finally, the reviewed studies did not examine or clarify the presence of complex PTSD, which is associated with distinct brain abnormalities (Marinova & Maercker, 2015). Overall, a great number of studies did not examine or control for these factors and it remains unclear whether their findings were actually derived from the PTSD symptoms.

Moreover, several methodological shortcomings and differences could have played a role in the variability of the findings. Particularly, the majority of studies on neurobiology had small sample sizes that might not be sufficient to detect significant effects. Furthermore, there are various MRI analysis techniques with different levels of sensitivity and specificity. For instance, it is suggested that automatic volumetric analysis might fail to detect subtle differences in brain structures compared to manual segmentation (Lindauer, Vlieger, et al., 2004). There is also a lack of research on brain network activity in PTSD under ongoing threat that could provide a broader picture of brain functioning. Additionally, studies on cortisol varied in type, time, and number of cortisol measurements. Most of the studies used multiple measurements of cortisol, but the specific time points varied significantly. These time differences could be responsible for the inconsistent findings on cortisol levels in PTSD and we should be very cautious when we compare the results of different studies. Most importantly, there is a lack of research that directly compares PTSD after ended trauma and PTSD under ongoing threat and, thus, no definite conclusions about their differences may be drawn.

### Clinical implications for treatment

The differences as well as the similarities between PTSD after ended trauma and PTSD under ongoing threat are crucial for clinical practice. The major difference between PTSD under ongoing threat and PTSD after ended trauma is the fact that the former is characterized by a continuous and probably unavoidable stressful and traumatic environment that fuels the PTSD. Particularly, a recent meta-analysis suggested that early administration of hydrocortisone after a traumatic event is effective in the prevention of PTSD (Sijbrandij, Kleiboer, Bisson, Barbui, & Cuijpers, 2015). Glucocorticoid administration is assumed to diminish the retrieval of traumatic emotional memories and promotes the extinction and inhibitory fear learning (Sijbrandij et al., 2015). Hydrocortisone augmentation leads to greater retention to treatment in PTSD patients with finished trauma, which in turn results in symptom improvement (Yehuda et al., 2015). However, it may not be helpful to administer even more cortisol to patients with PTSD under ongoing threat who already exhibit increased endogenous cortisol levels.

### Future research

This review presents the limited evidence on PTSD under ongoing threat and underscores that the findings are sparse and do not provide a clear image of the differences between PTSD under ongoing threat and PTSD after ended trauma. Further research may disentangle these differences and explore the substantially understudied population of individuals with PTSD under ongoing threat. It would be beneficial for future research to directly compare the neurobiology and neuroendocrinology of individuals with PTSD after ended trauma and PTSD under ongoing threat in order to draw reliable conclusions. Furthermore, it is crucial to take into account several covariates such as comorbidity, history of childhood abuse and methodological limitations in order to unravel the independent effect of PTSD. There is also a great need for randomized controlled trials that will investigate whether and how we could provide effective treatment for PTSD under ongoing threat. By revealing the similarities and differences between PTSD after ended trauma and PTSD under ongoing threat, we will be able to shed light on the different manifestations of PTSD and explain some of the inconsistent findings in brain patterns and HPA functioning in PTSD. Overall, this review underscores that the findings on PTSD under ongoing threat are limited, inconsistent, and with methodological limitations and urges future studies to focus on this line of research in order to understand the characteristics of PTSD under ongoing threat and draw more definite conclusions.
